# Supplementary material for: Impact of flushing procedures on drinking water biostability and invasion susceptibility in distribution systems
Source: Appl Environ Microbiol. 2025 May 13;91(6):e00686-25. doi: 10.1128/aem.00686-25 (PMC12175504; doi:10.1128/aem.00686-25)
Supplement: Supplemental material — Figures S1 to S8; Table S1. [file aem.00686-25-s0001.docx]

**Supplementary information of:**

**Impact of flushing procedures on drinking water biostability and invasion susceptibility in distribution systems**

**Fien Waegenaar^1,2^, Thomas Pluym^1,2^, Elise Vermeulen, Bart De Gusseme^1,2,3^, Nico Boon^1,2*^**

^1^Center for Microbial Ecology and Technology (CMET), Department of Biotechnology, Ghent University, Coupure Links 653, B-9000 Gent, Belgium.

^2^Center for Advanced Process Technology for Urban Resource Recovery (CAPTURE), Frieda Saeysstraat 1, B-9000 Ghent, Belgium.

^3^Farys, Department Innovation Water – R&D, Stropstraat 1, B-9000 Gent, Belgium.

*Correspondence: [nico.boon@ugent.be](mailto:nico.boon@ugent.be)


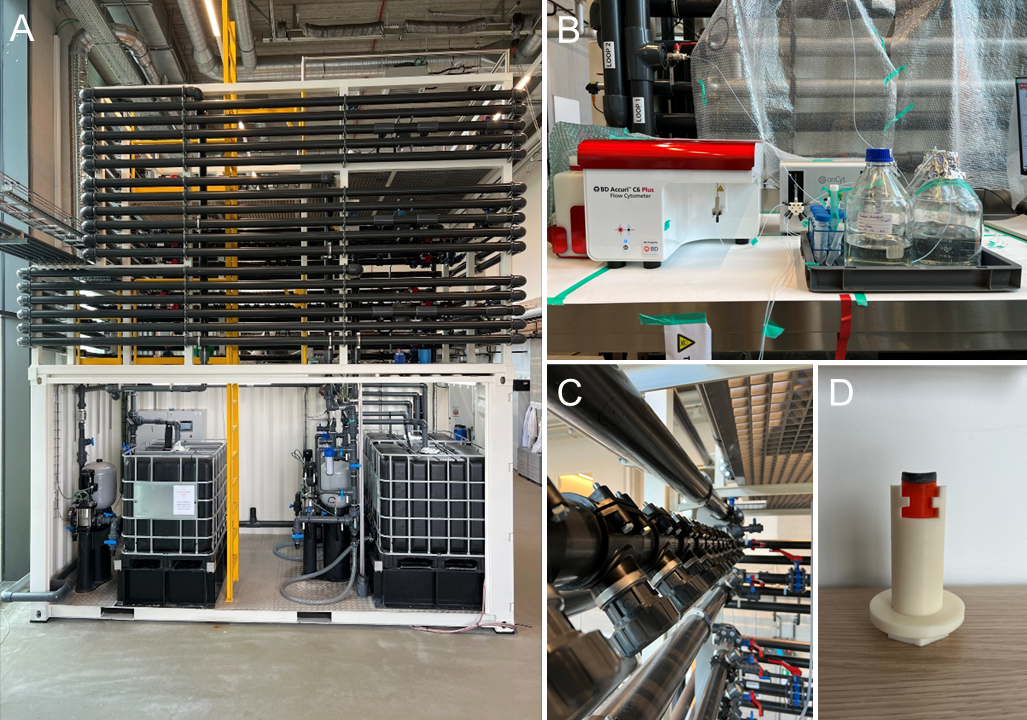


**Fig. A1: (A)** The drinking water distribution pilot comprises three identical loops of 100m each, connected to a non-translucent IBC. The structure measures 5.2 m × 2.6 m. **(B)** Implementation of online microbial monitoring: An Accuri™ C6 Plus flow cytometer (left) is coupled with an onCyt© autosampler, facilitating automated sampling from the pilot and cleaning solutions (right). **(C)** Biofilm sampling involves the use of coupons for undisruptive examination. These coupons are installed on a pipe using a system designed to resist pressure. **(D)** The coupon when removed from the pipe. It consists of a white holder (3.4 cm of diameter, 9.5 cm long), whit a small insert (2.2 cm of diameter, 2.5 cm long) that can be placed within it. The top of this insert, which comes into contact with the water, is made of PCV-U, the same material as the pipes.


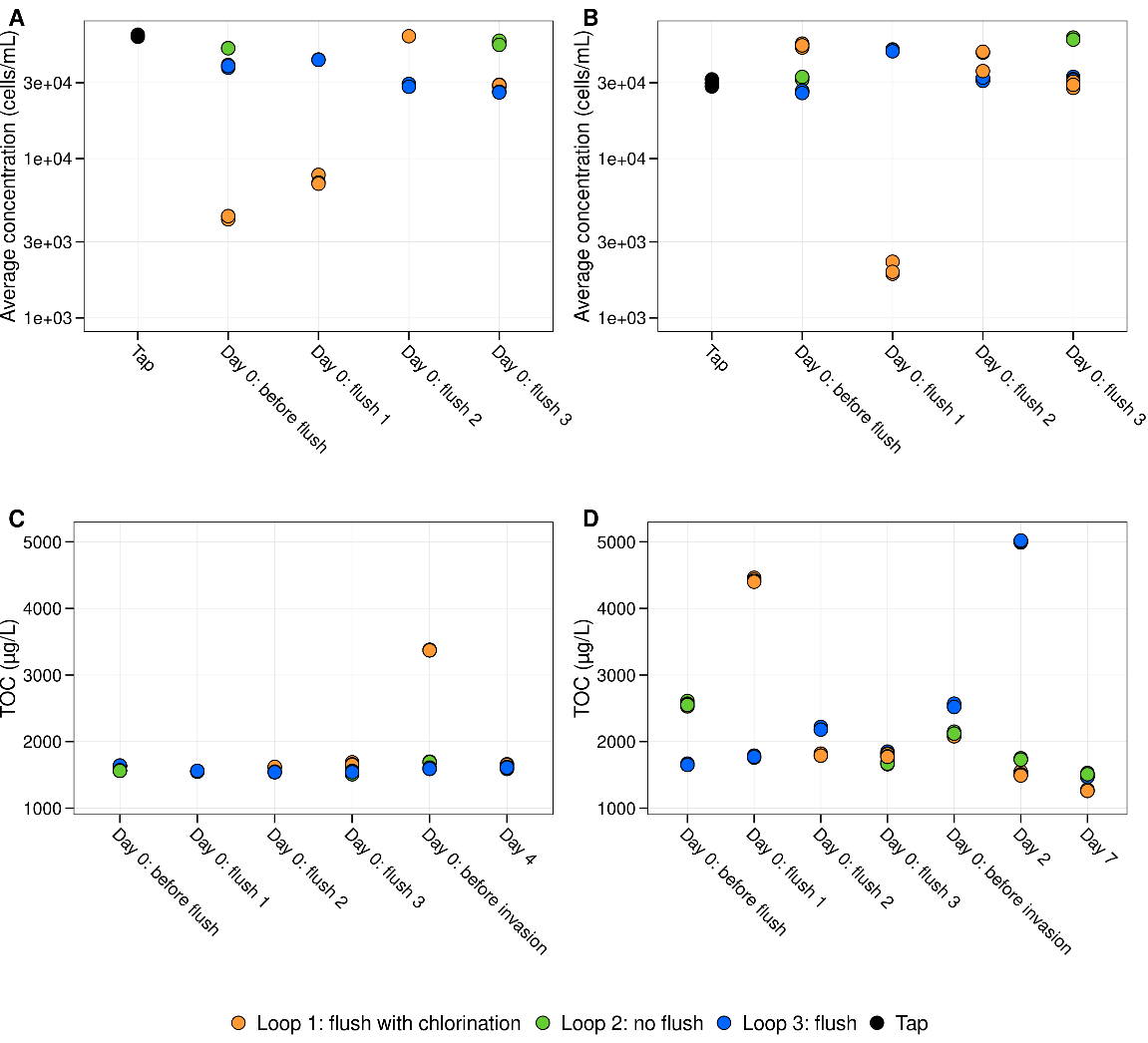


**Fig. A2**: Concentration (cells/mL) of the microbial community in the water fed to the pilot (black), loop 1 (orange), 2 (green), and 3 (blue) for water samples taken before, during and after the flushes during (**A**) the first experiment and (**B**) the second experiment. Total organic carbon (TOC) concentration in loop 1 (orange), 2 (green), and 3 (blue) for water samples taken before, during and after the flushes during (**C**) the first experiment and (**D**) the second experiment. Per timepoint, technical replicates (n = 3) were measured.


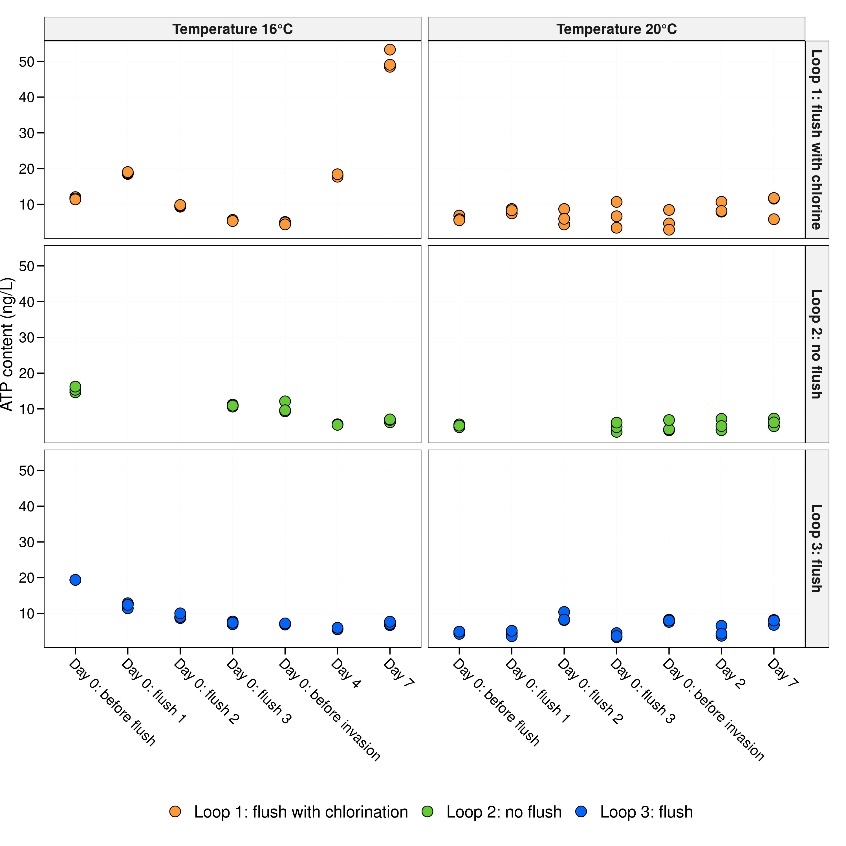


**Fig. A3:** ATP concentration (ng/L) of the microbial community in loop 1 (orange), 2 (green), and 3 (blue) for water samples taken before, during and after the flushes. Per timepoint, technical replicates (n = 3) were measured.


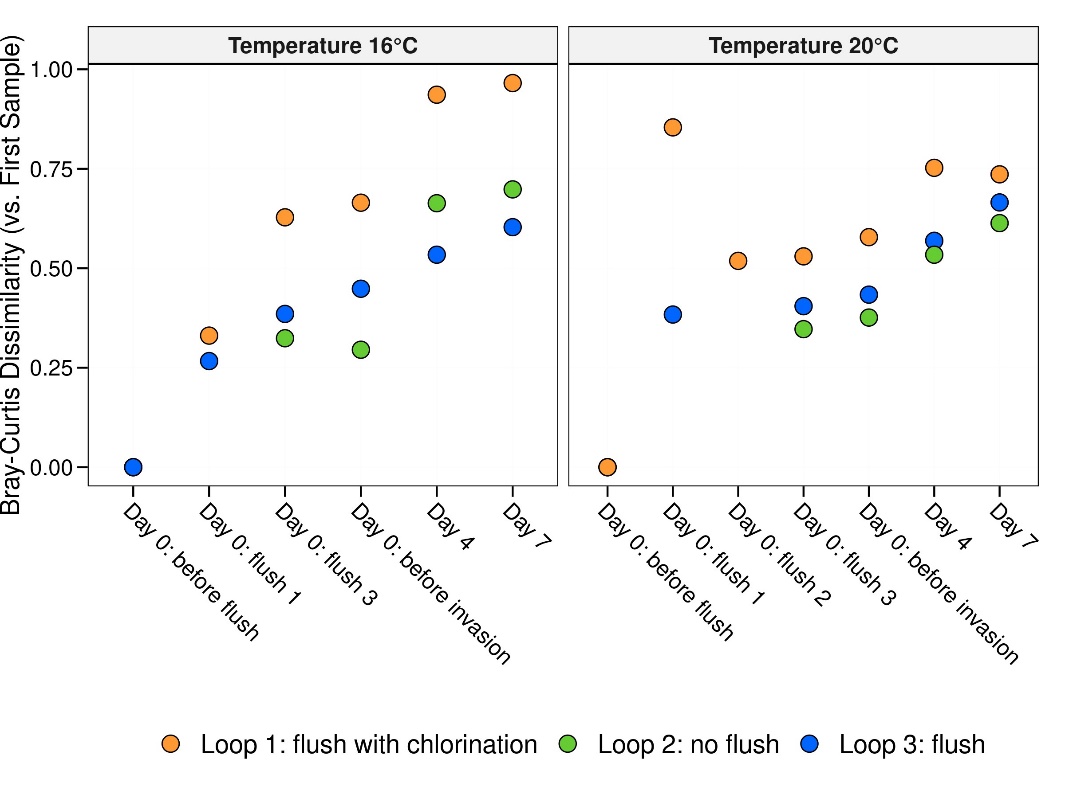


**Fig. A4:** Bray-curtis dissimilarity values per timepoint of the bacterial community in loop 1 (orange), loop 2 (green), and loop 3 (blue) when the water temperature was 16°C and 20°C. Bray-curtis dissimilarity values were calculated based on the 16S rRNA gene-base amplicon sequencing results.


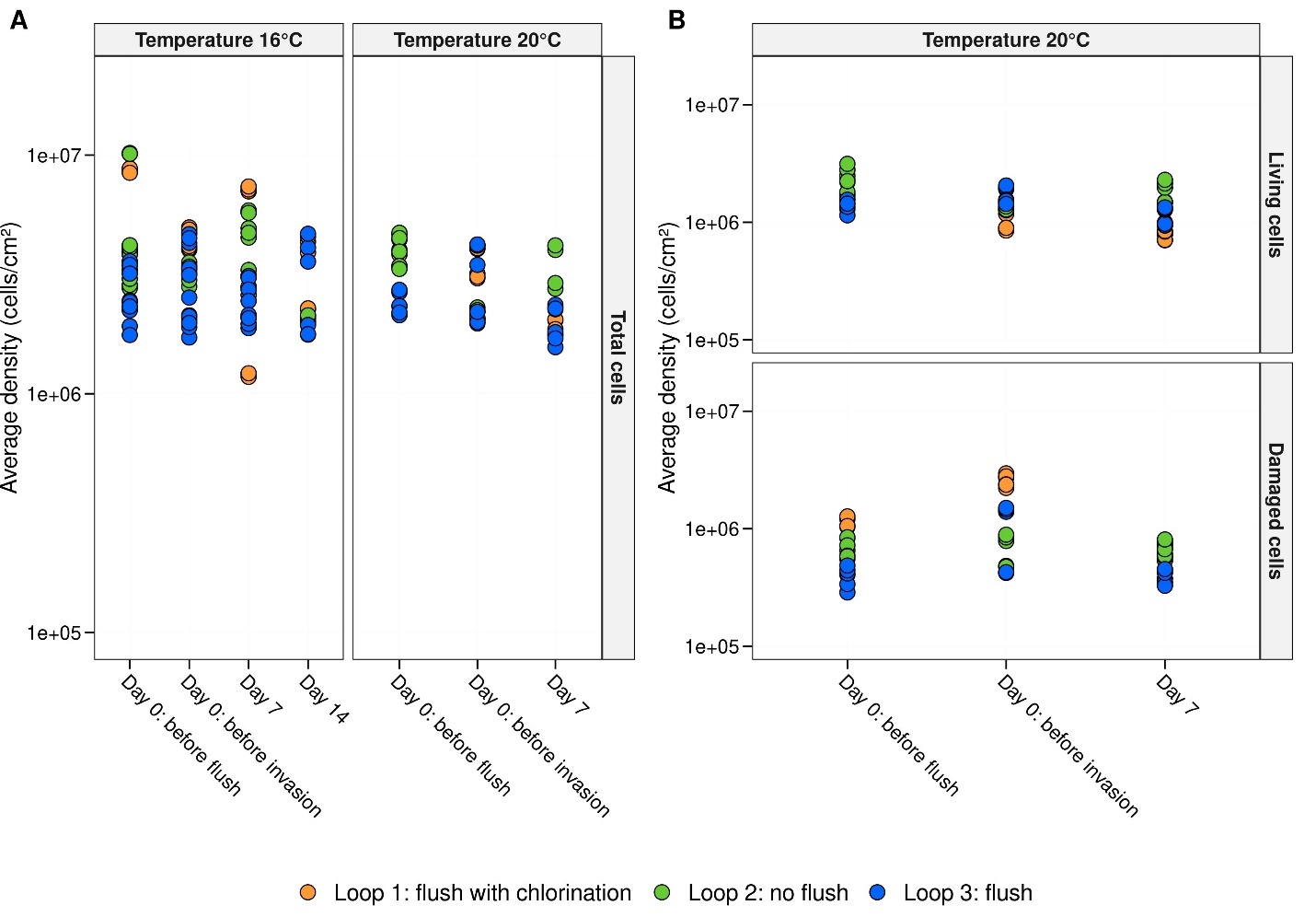


**Supplementary Fig. A5: (A)** Average total cell concentration (cells/cm²) per timepoint of the bacterial community in loop 1 (orange), loop 2 (green), and loop 3 (blue) when the water temperature was 16°C and 20°C. **(B)** Live-dead analyses to measure intact cell counts (cells/cm²) and damaged cell counts (cells/cm²) per timepoint of the bacterial community in loop 1 (orange), loop 2 (green), and loop 3 (blue) when the water temperature was 20°C.


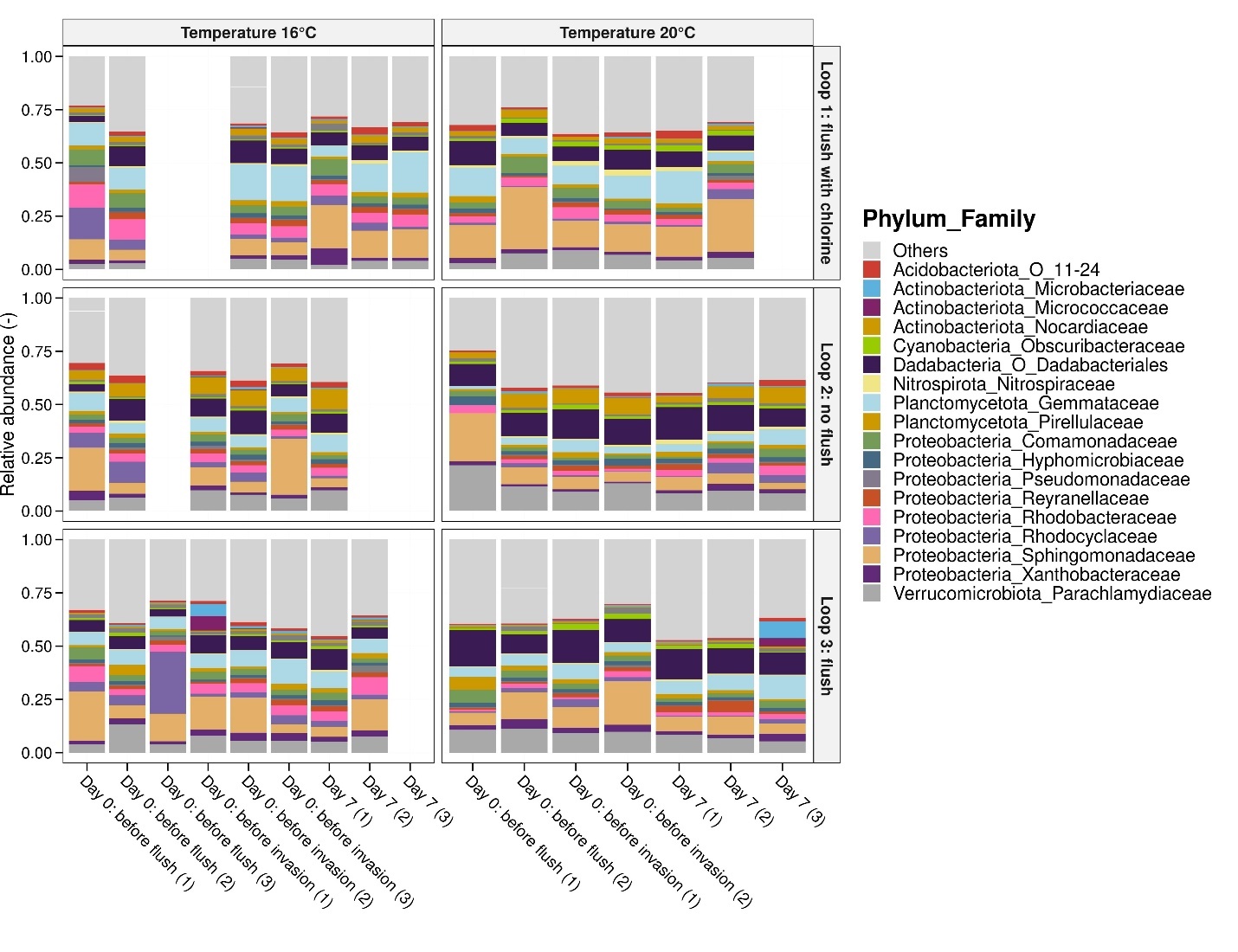


**Fig. A6:** Relative abundances of the 18 most abundant families of the biofilm before the flush, after the flush and after 7 days. Biological replicates (n = 2 or n = 3) were collected at each timepoint. Instances where no bar plot is presented correspond to samples with insufficient DNA for analysis.


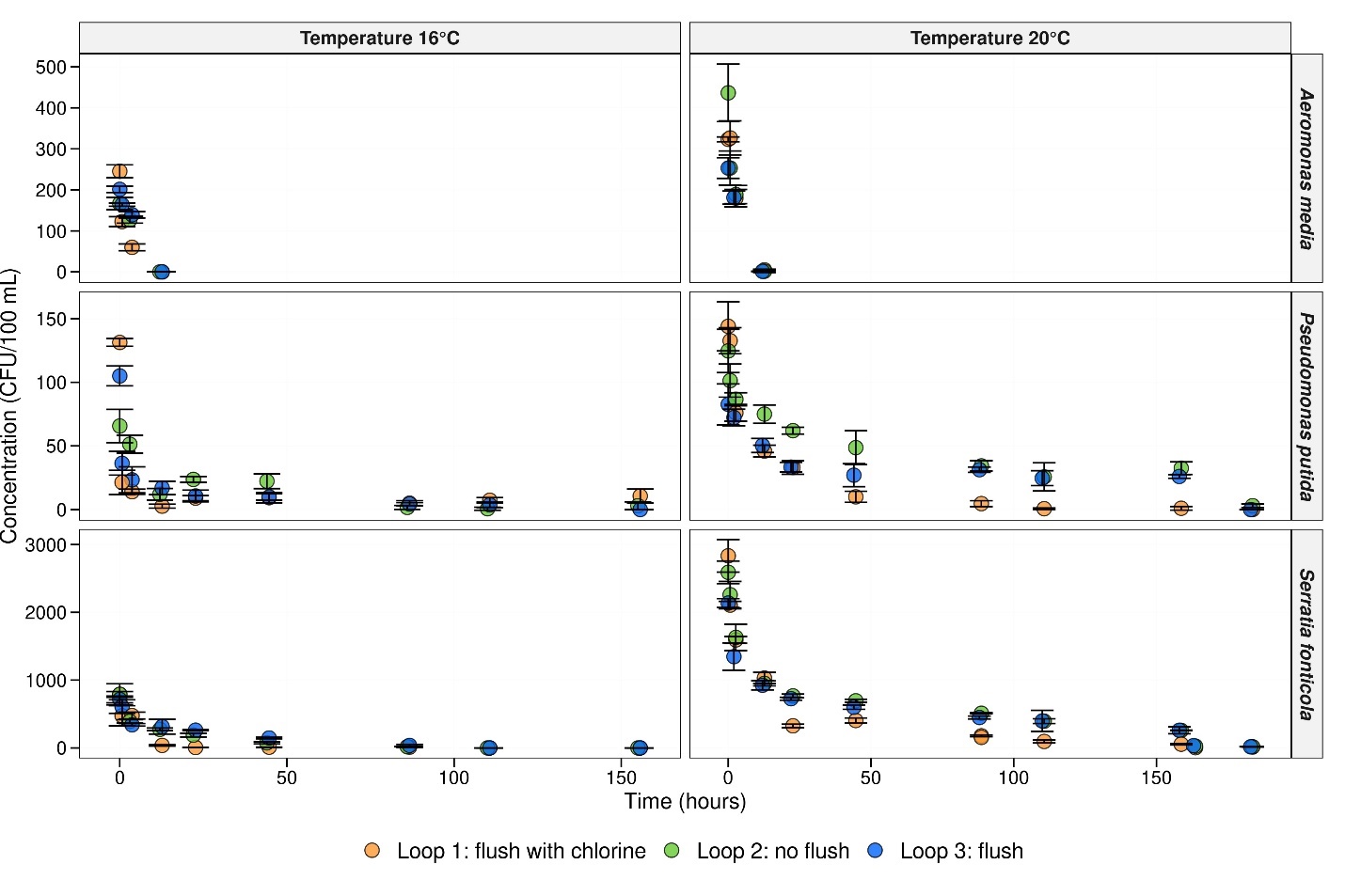


**Fig. A7:** Average concentration (CFU/100 mL) in function of time (hours) for each invader at each temperature scenario in loop 1 (orange), loop 2 (green), and loop 3 (blue). Per timepoint, biological replicates (n = 3) were taken and corresponding error bars are shown in black.


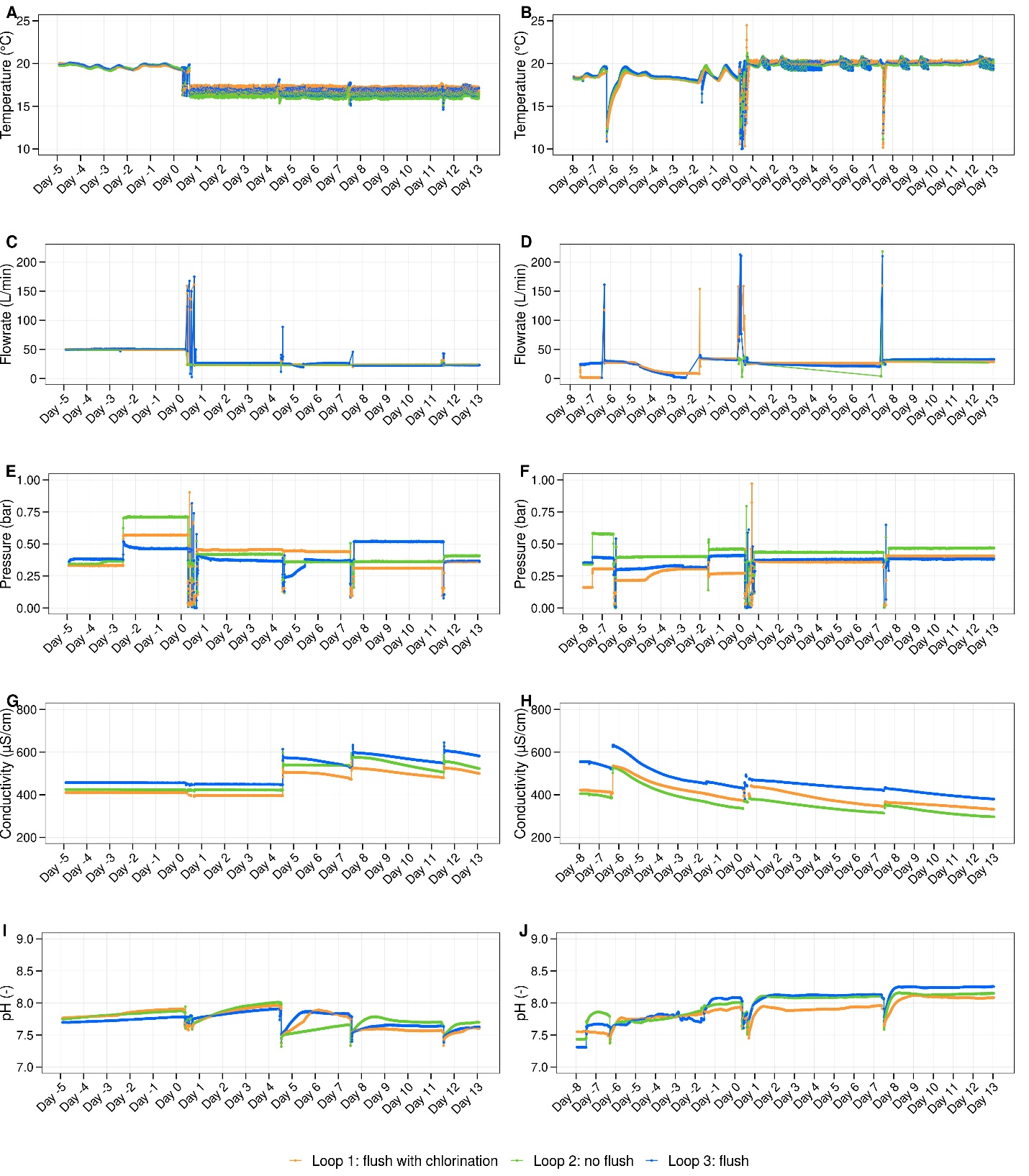


**Fig. A8:** (A, C, E, G, I) represent the physical parameters for the first experiment conducted at 16°C. (B, D, F, G, J) represent the physical parameters for the second experiment conducted at 20°C. The parameters are shown as a function of time for loop 1 (orange), loop 2 (green), and loop 3 (blue). Measurements were automatically taken every 5 minutes.

**Table A1:** Water quality parameters at the beginning and end of the experiment with the corresponding legislative norm, measured by Farys^1^. ‘-‘ indicates ‘not determined’ and ‘NAC’ means ‘non abnormal changes’.

| **Parameter** | **Observations of water fed to the pilot** | **Drinking water norm** |
| --- | --- | --- |
| **Source** | Surface water | / |
| **Color** | Absent | Absent |
| **Turbidity** | Absent | Absent |
| **Odor** | Absent | Absent |
| **Taste** | Absent | Absent |
| **Temperature (°C)** | 20.1 | 25.0 |
| **pH (20°C)** | 8.0 | 6.5 – 9.2 |
| **Conductivity (μS/cm at 20°C)** | 554 | 2500 |
| **Total hardness (°F)** | 17.7 | 67.5 |
| **Chloride (mg/L)** | 69 | 250 |
| **Nitrate (mg NO_3_/L)** | 2.73 | 50.0 |
| **Nitrite (mg NO_2_/L)** | <0.015 | 0.50 |
| **Ammonia (mg NH_4_/L)** | <0.050 | 0.50 |
| **Fluoride (mg/L)** | 0.41 | 1.5 |
| **Free chlorine (µg/L)** | <60 | 250 |
| **Alkalinity (mg/L)** | 162 | - |
| **Saturation-index** | 0.38 | >-0.50 |
| **Sulfate (mg/L)** | 64 | 250 |
| **Ortho-phosphate (µg P2O5/L)** | <15 | - |
| **Total coliforms (/100 mL)** | 0 | 0 |
| **Escherichia coli (/100 mL)** | 0 | 0 |
| **Enterococci (/100 mL)** | 0 | 0 |
| ***Clostridium perfringens* (/100 mL)** | 0 | 0 |
| **Counting colonies at 22°C (/mL)** | 4 | NAC |
| **Aluminium (µg/L)** | 53 | 200 |
| **Iron (µg/L)** | <10 | 200 |
| **Lead (µg/L)** | 1.6 | 10 |
| **Manganese (µg/L)** | <2.0 | 50 |
| **Nickel (µg/L)** | <5.0 | 20 |
| **Sodium (mg/L)** | 52 | 200 |
| **Potassium (mg/L)** | 4.8 | - |
| **Calcium (mg/L)** | 55 | 270 |
| **Magnesium (mg/L)** | 9.2 | 50 |
| **Bromate (µg/L)** | <1.0 | 10 |
| **Chlorate (µg/L)** | 120 | 250 |
| **Chlorite (µg/L)** | <1.0 | 250 |
| **Non-purgeable organic carbon (m/L)** | 1.6 | - |

1. emis, V. *Compendium Voor de Monsterneming, Meting En Analyse van Water*. https://emis.vito.be/nl/erkende-laboratoria/water-gop/compendium-wac (2020).
